# Assets and Challenges to Recruiting and Engaging Families in a Childhood Obesity Treatment Research Trial: Insights From Academic Partners, Community Partners, and Study Participants

**DOI:** 10.3389/fpubh.2021.631749

**Published:** 2021-02-22

**Authors:** Donna-Jean P. Brock, Paul A. Estabrooks, Maryam Yuhas, Jonathon A. Wilson, Danielle Montague, Bryan E. Price, Kenya Elliott, Jennie L. Hill, Jamie M. Zoellner

**Affiliations:** ^1^Public Health Sciences, School of Medicine, University of Virginia, Charlottesville, VA, United States; ^2^College of Public Health, University of Nebraska Medical Center, Omaha, NE, United States; ^3^Department of Nutrition and Food Studies, Syracuse University, Syracuse, NY, United States; ^4^City of Danville Parks and Recreation, Danville, VA, United States; ^5^Piedmont Access to Health Services, Danville, VA, United States

**Keywords:** recruitment, engagement, retention, attendance, childhood obesity treatment, community based participatory research

## Abstract

**Background:** There is need for the childhood obesity treatment literature to identify effective recruitment and engagement strategies for rural communities that are more likely to lack supportive infrastructure for healthy lifestyles and clinical research relative to their urban counterparts. This community case study examines recruitment and engagement strategies from a comparative effectiveness research (CER) trial of two family-based childhood obesity (FBCO) treatment interventions conducted in a medically underserved, rural region. Guided by a Community Based Participatory Research (CBPR) and systems-based approach, the primary aim was to analyze interviews from academic partners, community partners, and parent study participants for recruitment and engagement assets, challenges, and lessons learned.

**Methods:** Over the 3-year lifespan of the study, researchers conducted 288 interviews with Community Advisory Board members (*n* = 14), Parent Advisory Team members (*n* = 7), and study participants (*n* = 100). Using an inductive-deductive approach, interviews were broadly coded for recruitment and engagement assets, challenges, and recommendations; analyzed for descriptive sub-coding; and organized into stakeholder/organization and participant level themes. Codes were analyzed aggregately across time and examined for differences among stakeholders and parent study participants.

**Results:** Adherence to CBPR principles and development of strong community partnerships facilitated recruitment and engagement; however, variability in recruitment and engagement success impacted partner confidence, threatened outcome validity, and required additional resources. Specifically, assets and challenges emerged around eight key needs. Three were at the stakeholder/organization level: (1) readiness of stakeholders to conduct CBPR research, (2) development of sustainable referral protocols, and (3) development of participant engagement systems. The remaining five were at the participant level: (1) comfort and trust with research, (2) awareness and understanding of the study, (3) intervention accessibility, (4) intervention acceptability, and (5) target population readiness. Future recommendations included conducting readiness assessments and awareness campaigns, piloting and evaluating recruitment and engagement strategies, identifying participant barriers to engagement and finding a priori solutions, and fostering stakeholder leadership to develop sustainable protocols.

**Conclusion:** Collective findings from multiple perspectives demonstrate the need for multi-leveled approaches focusing on infrastructure supports and strategies to improve stakeholder and participant awareness of, and capacity for, recruiting and engaging medically underserved, rural families in a FBCO CER trial.

## Introduction

Recruitment and engagement challenges are well-documented in childhood obesity treatment studies (1–8). Poor recruitment negatively impacts internal and external validity (9) whereas low engagement may diminish intervention effectiveness in reducing child weight status (4, 10). The value of disseminating practical lessons on recruitment and engagement to support later clinical obesity treatment programming is clear, but often under-reported and under-investigated in the literature (11, 12). This gap in the literature is more apparent for rural communities (13) that have unique barriers relevant to recruitment and engagement [e.g., distrust and negative perceptions of research, geographic distances and low population density, limited access to technology, scheduling issues surrounding multiple jobs and non-traditional work shifts, and hindered ability to access resources (14–16)].

The current study attempts to expand this literature by examining recruitment and engagement (i.e., retention and participation) strategies from a comparative effectiveness research (CER) trial and family-based childhood obesity (FBCO) treatment intervention targeting a high need, medically underserved, rural region. Using a case study approach, researchers analyzed qualitative interview data across the 3-year study from three stakeholder groups that included academic partners, community partners, and parent study participants. The primary aims were to explore the assets and challenges to the various recruitment and engagement strategies attempted during the lifespan of the study and to highlight insights into effective practices and recommendations to help build sustainable systems to engage rural families in FBCO interventions.

## Context: Study Setting and Target Population

The study takes place in the Dan River Region (DRR), located in south central Virginia. It includes a small city, Danville, surrounded by large rural areas and small towns. The DRR is a federally designated, medically underserved area with challenging social determinants for poor health outcomes such as high poverty, unemployment, and minority concentrations (17, 18). Out of 133 counties, Danville city and Pittsylvania County are ranked at 127 and 87, respectively by the Robert Wood Johnson Foundation County Health Rankings (19). Among these statistics, adult obesity rate is 30% (city of Danville) and 31% (Pittsylvania County). While data for county level childhood obesity rates is limited, one local school district revealed rates three times higher than the state average (13%) (20). These high childhood obesity rates are exacerbated by disparities in healthy food access, built environment to promote physical activity, and access to effective treatment programs (21–23).

## Methods

### Study Background: Context Within the Larger CER Trial

The current study evolved from a preliminary Community-Based Participatory Research (CBPR) study in the DRR that adapted and piloted *i*Choose, an evidence-based FBCO (2013–2015) (24–28). As part of this pilot, researchers established a Community-Academic Advisory Board (CAB) as well as a Parent Advisory Team (PAT) made of former *i*Choose parent participants. Continued collaboration with the CAB and PAT resulted in a Patient Centered Outcomes Research Institute (PCORI) contract (2017–2020) to conduct a randomized CER comparing two different FBCOs in the DRR: *i*Choose (high intensity) and *Family Connections* (low intensity). The rationale and study protocol are published elsewhere (29).

This CER included three cohorts of families, recruited and randomized over the 3-year study period. In brief, *i*Choose was adapted from an evidence-based FBCO, Bright Bodies (24, 27, 30–32), targets the parent/child dyad, and includes ~64 contact hours (i.e., family classes, family exercise sessions, interactive voice response support calls, child newsletters). *Family Connections* (33) was adapted from Golan's Home Environmental Change model (34), targets parents as the agents of change, and includes approximately five contact hours (i.e., parent classes, interactive voice response support calls, promotion of physical activity without structured exercise sessions). The target population for this study were families within the DRR that had children with a BMI percentile of 85% or greater.

**Figure 1:**
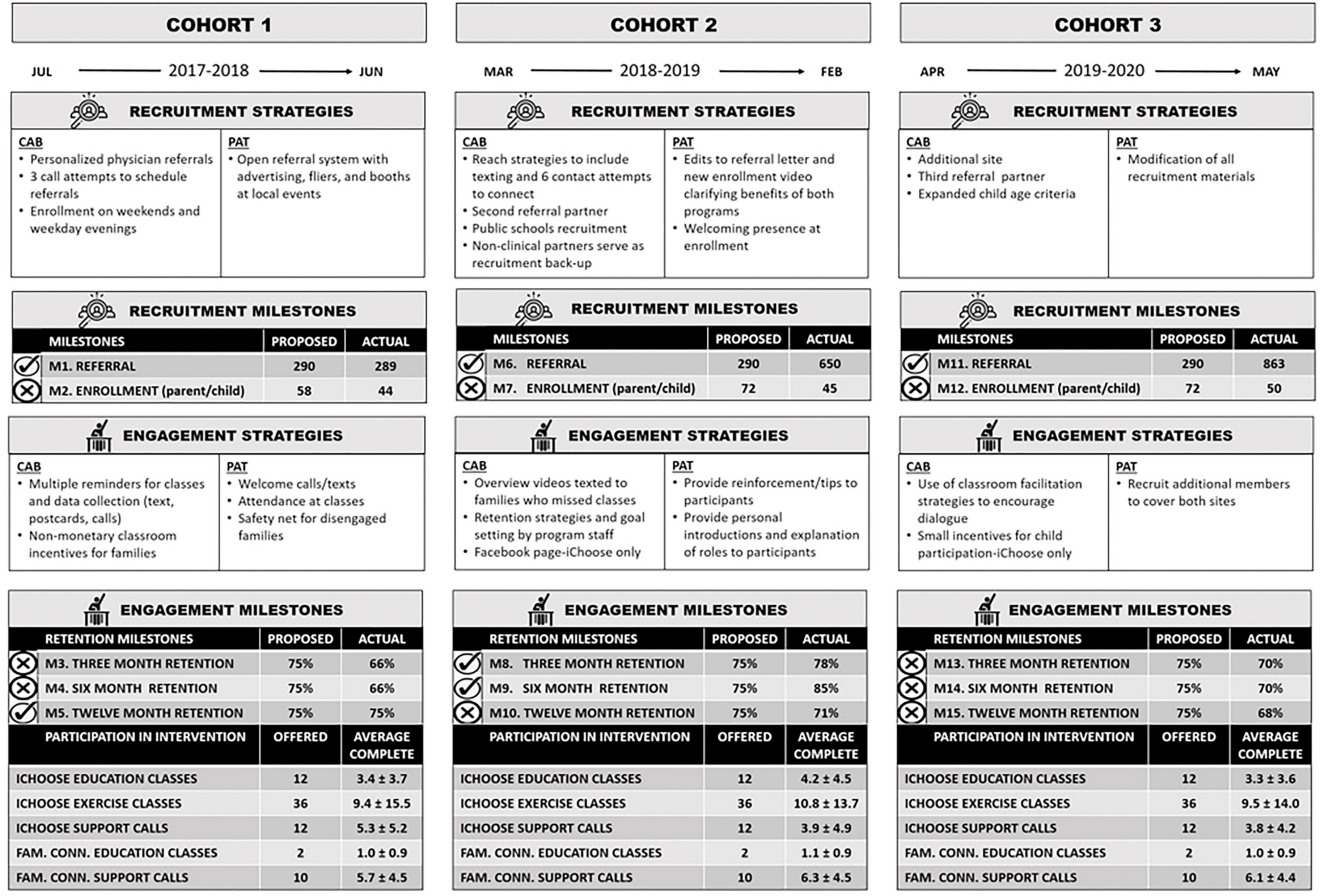
Timeline of milestones and data collection events with recruitment and engagement strategies^a^ for milestone achievement. ^a^Strategies indicate cumulative efforts, with additional strategies at cohorts 2 and 3 adding to those from cohort 1, respectively.

The decision to focus on recruitment and engagement within this case study was driven from consistent struggles to meet milestones in these areas, despite numerous evidence-based practices and adaptations to address challenges. As illustrated in Figure 1, referral milestones were met or exceeded; however, enrollment milestones consistently fell short (i.e., enrollment rates for Cohorts 1–3 were 76, 63, and 70% of milestones targets). Of referrals that did not enroll, a large proportion (40–56%) were unreachable via phone while about one-third verbally declined. The main reasons for declining included lack of interest and time conflicts. Similarly, six of the nine retention milestones, while close to success, were not achieved (see Figure 1). Within both interventions, participation rates were lower than anticipated. Overall, average participation in *i*Choose was approximately four of the 12 classes and one-third of support calls. In contrast, average *Family Connections* participation was about one of two classes and two-thirds of support calls.

### Study Design

This case study focused upon recruitment and engagement data garnered from qualitative interviews collected annually over the 3-year project with academic CAB partners and community CAB and PAT partners, and at each follow-up assessment with parent study participants. The University of Virginia's Institutional Review Board approved this research. Prior to participating, all interviewees provided written consent.

### Recruitment and Engagement Procedures: A CBPR Approach

As seen in Figure 1, a CBPR approach was used to develop and implement recruitment and engagement strategies. As such, these strategies were carried out by CAB and PAT partners according to their expertise, resources, and access to potential participants. Deliberate selection of community CAB partners included clinical representatives from the Southern Virginia Health (SOVAH) Pediatrics and the Piedmont Access to Health Services (PATHS); program delivery representatives from Danville Parks and Recreation; and other community organizations with missions to improve the health of families in the DRR (e.g., Danville Public Schools). Academic CAB partners consisted of researchers from the University of Virginia, Virginia Tech, and the University of Nebraska. The CAB's primary functions were to determine logistics, provide expertise and manpower, and resolve issues surrounding recruitment, implementation, retention, and data collection. More specifically, clinical partners were invited to the CAB to develop referral and recruitment protocols, programming partners to lead in implementation and engagement, and academic partners to manage research protocols and data collection, analysis, and dissemination. Clinical and community partners were subcontracted for their role on this project.

As former participants in either the pilot or current study, PAT partners represented families targeted by the FBCO efforts. They took leadership over open source recruitment (i.e., community promotion), engaged with families and program facilitators during classes, and provided support via phone and texts to participants. PAT membership fluctuated from five to seven members, with all receiving a monthly stipend of $100–$200 depending upon activities and responsibilities.

### Demographics of Enrolled Study Participants in the CER Trial

Interested families were screened for eligibility criteria (i.e., child BMI percentile ≥85, English speaking, and no child contraindications for participation). The majority of the 139 enrolled children (70%) and 128 enrolled parents (75%) were obese. Over half the children were black (45%) or other minorities (8%), lived in single parent homes (55%), and were enrolled in Medicaid (62%). About one-third of parents (average age of 39) had a household income of < $20,000 (29%), were unemployed (32%), had a high school diploma or less (30%), and a less than adequate health literacy score (29%).

### Interview Procedures and Instruments

During the study, a total of 288 interviews were conducted with five academic CAB partners, nine community CAB partners, seven PAT partners, and 100 parent study participants (Table 1).

**Table 1:** Sample size for academic CAB partners, community CAB and PAT partners, and parent study participants.

**Key stakeholder groups/Study participants**	**First interview/3 m follow-up**	**Second interview/6 m follow up**	**Third interview/12 m follow-up**	**Total interviews^**a**^**
Academic CAB partner	5	5	5	15
Community CAB partner	9	8	5	22
PAT partner	5	5	7	17
Parent study participant	86	88	60	234

a *Analysis of data is aggregated across time so that the ultimate unit of analysis is 9 for community CAB members, 5 for academic CAB members, 7 for PAT members, and 100 for parent study participants*.

CAB and PAT partners participated in three annual interviews conducted and recorded by an independent evaluator using a semi-structured interview script. The script was developed from a capacity framework guided by CBPR principles (25, 26, 35). The framework constructs included communication, problem solving, development of community power, as well as roles and processes of the CAB and PAT. PAT members were asked about their role in recruitment and engagement at each interview, while CAB members were specifically asked these questions at their third interview. Interviews lasted 30–90 min and were transcribed verbatim.

Parent study participants from each cohort participated in face-to-face interviews at 3-, 6-, and 12-month follow-up assessments. Trained researchers probed about recruitment and randomization processes and intervention adherence, usefulness, and satisfaction. Children were also interviewed at these assessment points; however, the focus of these interviews was more upon individual and family behavior changes and general satisfaction with the program (iChoose only) than with recruitment and engagement facilitators and obstacles. Their data added little value to the purpose of this paper and therefore is not included. Incentives of $25 for 3-month and $50 for the 6-and 12-month follow-ups were offered to study participants. Interviews lasted 10–20 min. Researchers summarized responses on paper and recorded verbatim responses when possible.

### Data Analysis

Analysis of the qualitative data were conducted in two phases (36). In the first phase, researchers used an inductive approach to code data for recruitment and engagement meaning units (36–38). This included text searches for terms, processes, strategies, and outcomes related to recruitment and engagement. Researchers identified these references as either assets, challenges, or recommendations. Coders familiar with the study worked in teams of two. They first coded interviews independently and then came together to reconcile disagreements and refine codes. The resulting codebook and interview content were entered into NVivo (QSR International, 1.2). Researchers content analyzed primary codes for descriptive sub-codes related to the assets, challenges, and recommendations (36). These sub-codes underwent the same reconciliation and data entry processes described above.

Phase two analysis was completed by the Principal Investigator and Study Coordinator. Together they used a deductive approach to thematically categorize codes into eight higher level study needs that present at the stakeholder/organization and the individual levels (36, 37). Data were examined aggregately across the study years and examined for differences in perspectives among the stakeholders and study participants.

## Results

Supplementary Table 1 reports assets and challenges for each of the stakeholder/organization and participant level needs, including prevalence across stakeholders and study participants. Tables 2, 3 summate recommendations to address the needs.

**Table 2:** Recommendations for stakeholder/organization level needs.

**Stakeholder/Organization level**
1. **Improve readiness of the stakeholders to conduct CBPR research**
• Normalize failures as part of the research process and encourage problem solving • Plan for adjustments in collaboration due to introduction of new members • Demonstrate connection/overlap between organization and study missions • Discuss conflicts between partners up front • Clarify roles and responsibilities and how they inter-connect • Ensure partners understand the intervention and role model healthy lifestyle behaviors
2. **Develop sustainable referral protocols**
• Create eligibility criteria that is not too exclusive to the needs of the community • Encourage and promote leadership in recruitment activities • Include providers in the protocol with an emphasis on point of contact recruitment • Expand recruitment roles and create backup protocols to address unanticipated events • Expand resources through strategic partnering/networking • Tie budget to reflect organizational capacity to carry out recruitment roles • Regularly evaluate, update, and reflect upon recruitment findings • Link partner referral pools with realistic estimates of family enrollment • Adapt recruitment strategies as necessary based upon findings • Transparency in partners abilities to follow through on tasks • Develop a safe environment to report obstacles and lack of progress • Create agreed upon channels of communication to report recruitment progress • Provide transparent estimates of time allocation for partner recruitment efforts
3. **Develop feasible and sustainable engagement protocols**
• Create feedback loops to share best practices and challenges for engaging participants • Provide adequate compensation for efforts and strategically partner to grow resources • Regularly evaluate, update, and reflect upon engagement practices • Provide cross training to program facilitators so back-up protocols are in place • Encourage leadership in engagement by recognizing partner efforts and successes • Establish clear engagement roles and responsibilities for all stakeholders • Link strategies to successful re-engagement and track reasons for dis-engagement • Develop regular training to review content and discuss strategies and challenges

**Table 3:** Recommendations for participant level needs.

**Participant level**
1. **Improve target population's level of comfort and trust with research**
• Educate the target population on the role of research in combatting childhood obesity • Link childhood obesity rates and causal models to the purpose of the study • Develop partnerships with community members from trusted local organizations
2. **Improve target population's accessibility to the intervention**
• Assess attendance barriers prior to enrollment and throughout the study • Choose sites that are central to the community and located by public transportation • Use additional sites and asynchronous approaches to deliver curriculum • Offer drop-in options/rolling enrollment for participation • Provide multiple day and time slots for the program to accommodate schedule conflicts • Anticipate seasonal barriers when finalizing the program calendar • Provide child care for families that need it • Offer incentives to offset costs of participation
3. **Improve target population's awareness and understanding of the study**
• Market strategically to communities with higher needs/risks • Create and pilot compelling marketing messages, materials, and strategies • Review materials for health literacy using universal precautions • Apply CBPR principles to recruitment (e.g., co-learning) • Use multiple modalities to reach participants (e.g., television, radio, and social media) • Anticipate regulatory obstacles and work to compromise with institutions like the IRB • Seek marketing expertise in planning a campaign • Create an adequate marketing budget
4. **Improve target population's acceptance of the intervention**
• Provide additional support to maintain healthy lifestyle changes • Address the collective and individual needs of the families with interactive curriculum • Use stakeholder and participant feedback to inform solutions to engagement barriers • Expand eligibility criteria to be more inclusive • Offer incentives to increase participant compliance • Welcome all family members to participate and create family activities to engage in • Allow families to choose the intervention that fits their needs • Ensure that facilities are appropriate, comfortable, and welcoming • Measure family progress and provide support to those struggling to make changes
5. **Improve target population's readiness to engage in a childhood obesity treatment study**
• Create a childhood obesity awareness campaign within the targeted community • Provide clinician training on messaging of child weight status during appointments • Assess readiness of participants to make changes prior to enrollment • Emphasize the benefits of the study for participants and their community • Encourage participants on their progress and find solutions for setbacks • Have participants set study engagement goals • Share individual health metrics during assessments to demonstrate progress

### Stakeholder/Organization Level Needs

At the stakeholder/organization level, three thematic needs emerged: (1) improve readiness of stakeholders to conduct CBPR research, (2) develop sustainable participant referral protocols, and (3) develop feasible and sustainable participant engagement protocols. As illustrated in Supplementary Table 1, CAB and PAT partners noted various assets to address these needs. These assets included partner commitment and dedication to the study along with a willingness to provide leadership; access to eligible participants through clinical partners; proactive development of systems to monitor recruitment and engagement and the flexibility to adapt strategies as necessary; cross training of staff as preparation for back-up in carrying out strategies; and comprehensive training for implementers. Conversely, identified challenges included community partners' lack of confidence in their research abilities and an over-reliance on academic members to lead in this area; difficulty navigating research regulations; disruptions in procedural continuity due to turnover in CAB or PAT membership; unanticipated difficulties that precluded a proactive response and arguably stretched partner resources; accumulation of recruitment and engagement strategies that made their individual impacts difficult to ascertain; and the consequences of partner time limitations on task follow-through.

Despite strong initial assets to meet stakeholder/organization level needs, the study under-performed with regard to milestones and expectations for recruitment and engagement. Suggestions to address these challenges included a number of already enacted strategies (see Figure 1), such as the development of back-up referral protocols and strategic partnering to address resource shortfalls, evaluation and reporting out of reach and process data, opportunities to discuss findings, and brainstorm new strategies when necessary (e.g., expanding age eligibility criteria), and development of additional protocols and training to implement new strategies (Table 2). Other recommendations seen in Table 2 stressed a need for greater emphasis and clarity in linking study goals with organizational missions, roles and responsibilities with goal advancement, and budget allocations with partner efforts. Also, the need to thoroughly orient new members to the study and their subsequent responsibilities was identified. Most importantly, recommendations included recognition of community partner efforts in recruitment and engagement to promote future integration of protocols into existing systems within their organizations.

### Participant Level Needs

At the participant level, five thematic needs for target population improvements emerged: (1) level of comfort and trust with research, (2) accessibility to the intervention, (3) awareness and understanding of the study, (4) acceptance of the intervention, and (5) readiness to engage in an obesity treatment study. As seen in the assets outlined in Supplementary Table 1, study partners made efforts to address childhood obesity needs in the community, participant barriers related to location and timing of the interventions, and cultural sensitivity within recruitment materials. They also worked to develop engaging curricula and strategies to motivate families to participate in the study. However, participant mistrust in the research process, along with time limitations, unexpected life events, communication challenges, and low health literacy countered these efforts. Furthermore, acceptability issues related to participant dissatisfaction with randomization and the “user experience” of the intervention, declining attendance, and slow goal attainment were seen as primary causes of study attrition. Cultural factors such as the lack of awareness of the childhood obesity crisis, acceptance of unhealthy child weight status as normal, and a community that does not prioritize health or encourage healthy lifestyle changes were seen as inhibiting overall readiness to engage.

Similar to the stakeholder/organization level needs, challenges outweighed assets and many of the recruitment and engagement milestones were not met. Regardless, existing strategies such as using community members from trusted local organizations and the targeted population to recruit and implement the study (see Figure 1) were seen as important to future efforts (Table 3). CAB and PAT members also recommended strengthened marketing strategies that include prior feedback from all stakeholders to counter target population distrust of research, unfamiliarity with study opportunities, and lack of readiness to engage in an obesity treatment study (Table 3). These marketing strategies could be made more efficient through strategically pinpointing the highest need groups within the targeted population. Additional recommendations outlined in Table 3 included addressing intervention accessibility with greater flexibility in attendance options and additional modalities for program implementation. To address acceptability issues, interviewees suggested replacing randomization with choice, expanding interventions to include highly desirable components, and opening enrollment to all interested family members regardless of BMI status. Others suggested that individual level assessments of readiness and participation barriers could be key in identifying successful strategies to improve retention.

### Similarities and Differences Among Academic CAB Partners, Community CAB and PAT Partners, and Parent Study Participants

With regard to stakeholder/organization level needs, academic CAB, community CAB, and PAT partners' perceptions of assets and challenges aligned similarly. However, there were notable difference in perceptions between these study stakeholders and the parent study participants for these needs. While parent study participants had opportunity to discuss issues surrounding their participation, they were much less likely than the community stakeholders to identify system level assets or barriers and largely kept their comments at the participant level. As noted later in the discussion, this disparity may also be a result of methodological differences in the intention and structure of the interview scripts in which study participants were not specifically prompted to reflect at the system level.

At the participant level, academic CAB partners were more likely than community CAB partners, PAT partners, and parent study participants to acknowledge target population's distrust of research. However, community CAB partners identified pathways to increase comfort through provision of interventions that clearly address key community burdens. All interviewee groups stressed challenges to accessibility and assets to intervention acceptability; however, CAB members mentioned challenges to acceptability less frequently than did PAT partners and parent study participants. Intervention acceptability, according to parent study participants, was particularly impacted by the level of satisfaction with randomization and intervention components. Finally, relative to enrolled parent study participants, CAB and PAT partners were less likely to perceive assets and more likely to perceive challenges to participant readiness to engage in a childhood obesity study.

## Discussion

Findings across the lifespan of our study demonstrated a pattern of recruitment and engagement challenges. These struggles were not fully anticipated by the CAB and PAT due to the application of evidence-based strategies that were previously successful with this target population (24) and additional community outreach and safety net infrastructure to guide recruitment and engagement strategies for participants (29). Because of the established participatory structure of the CAB and PAT and prior childhood obesity study (24–28, 35), barriers of the DRR were thought to be understood. However, the translation from a pilot study to a CER trial may have aggravated possible rural population barriers present in this target population. Thus, the complexity of the cumulative system-based challenges faced by this CER study provided an opportunity to examine our multi-perspective data for recruitment and engagement lessons pertinent to future FBCO interventions, especially in medically underserved rural areas.

### Lessons Learned and Future Implications

When considering the recommendations supported by our qualitative case study, it is clear that full system-changes are necessary to improve recruitment and engagement in similar childhood obesity studies being conducted in comparable health disparate regions. Potential changes to community and organizational structures that provide opportunities and access to these studies in a way that ensures broad exposure across the intended audience are likely to be the most impactful. While we note that participant readiness is both an asset (from parent study participant perspectives) and a challenge (from CAB participant perspectives), it is critical to avoid the assumption that the challenge of low recruitment is based on an unmotivated intended audience. As such, our recommendations include a strong focus on ensuring target population awareness and access to research, including trust building opportunities. Likewise, we focus on system changes that could influence norms related to how initial recruitment and sustained engagement can be supported at the research and community-system level.

*Lesson 1. Nurture CBPR principles that encourage co-learning and foster capacity building to conduct research*. The cornerstone of this study was partner readiness to conduct CBPR. Interviewees credited the CBPR approach in developing recruitment and engagement strategies reflective of community partner knowledge of the population and achieving higher milestone success than what might have been possible without this approach. The importance of community stakeholder engagement in research is supported in the CBPR literature (39–41). Although community partners may have been committed to the CBPR task of shared problem solving and decision making, building understanding of research protocols proved more challenging. Gaps in research knowledge can create tensions between academic and community partner priorities (42). For our study, the primary tension was balancing the researchers' need to follow protocols, stick to contract milestones, and maintain marketing regulations with community partners' beliefs that these elements hinder study acceptability. These findings emphasize the importance of co-learning in CBPR, specifically the need to take time to learn about the targeted population needs from community partners and research procedures from academic partners.

*Lesson 2. Prior to implementation, strategize for challenges identified by community stakeholders and prepare partners and systems to respond to unexpected obstacles*. While co-learning achieved through CBPR has the potential for responsiveness of all involved parties, it increases shared responsibility when things do not go as planned. Partners were confident in their knowledge of community challenges and how to address, yet this was based on a pilot study that was not a randomized control trial. As such, failure to fully meet recruitment and engagement milestones in this study was unexpected, sent the research team into “crisis” management mode, and negatively impacted the CAB and PAT's collective efficacy. Solutions to counter the lower than expected recruitment and engagement efforts focused on expanding CAB and PAT membership and deepening responsibilities. Support was forthcoming from committed partners willing to step into additional leadership roles and bring on new partners to provide extra resources. However, sufficient time to address partner discouragement, invest new partners in the vision of the project, and proactively prepare systems for additional challenges was not possible as the research team regressed to a reactive strategy implementation with the hope of finding a solid solution. The CBPR partnership readiness model recommends a continuous process of reviewing CBPR principles, building key competencies, and encouraging balanced dialogue between academic and community partners (43). As emphasized above, the recruitment and engagement assets attest to a high degree of adherence to these recommendations, particularly with regard to willingness to take leadership roles. However, collaboratively identifying and preparing for challenges, developing systems to orient and train new members, and assuring community partners of the normalcy of study setbacks could help strengthen overall readiness and resilience to obstacles.

*Lesson 3. Actively engage clinical partners to create systems for enrollment, adherence, and retention of study participants*. With regard to referral source, study participants indicated that they trusted their physicians and attributed their enrollment to provider referral. In our study, physicians identified eligible families through medical record review and mailed referrals rather than provided point of contact referrals. Furthermore, although physicians sent letters encouraging enrolled participants to return for follow-up assessments, they did not encourage adherence and retention in any formal, systematic capacity. Interviewees suggested that increased physician involvement in these capacities could be an important addition to a sustainable referral protocol. The benefits of provider interaction within weight management programs is supported in the literature (44). Our interviewees and the research literature both recommend physician training and consistent messaging on obesity within the clinic setting as an effective means to improve participants' readiness to engage in a FBCO study (44).

*Lesson 4. Create eligibility requirements that promote beneficence, particularly when the study is set in an under-resourced, rural community*. Stringent eligibility requirements may hinder building of sustainable referral and engagement protocols (45). While cohort 3 allowed younger children to enroll, the study protocol and treatment framework excluded children with normal BMI percentiles. Given the DRR health risk factors, it is possible that a prevention framework could better address the needs of this population. Research indicates that among communities where participants face numerous barriers to participation, elimination of a BMI eligibility requirement broadens the recruitment pool, allows for entire families to participate, and provides opportunity for additional recruitment partners (45–47).

*Lesson 5. Develop process evaluation plans for recruitment and engagement that can assess for success of individual strategies and changes following adaptations*. Our study closely tracked the pathway for enrollment, engagement/disengagement, and retention. CAB and PAT members recommended evaluation as vital in understanding when strategies needed to be adapted. This is consistent with health behavior change study reviews that call for more rigorous reporting on recruitment and engagement practices (41). Thoughtful evaluation would better link recruitment and engagement strategies to accrual and retention outcomes and help inform best practices (45).

*Lesson 6. Leverage feedback from community partners in developing clear, appropriate, and understandable messaging about the study to the intended audience*. Communications informing the target population about the purpose and processes of the research helps foster trust and encourage participation (48–50). Messaging about the study during enrollment should emphasize benefits in easy to understand language and explain procedures like randomization (41, 46). For example, community CAB members identified that intervention benefits were obscured under technical consent procedures and that predetermined intervention preferences and mistrust of randomization processes were causing dissatisfaction and potentially influencing early dropout. Subsequently, they worked with academic partners to develop a brief video describing the study. By cohort 2, the video was viewed by study participants prior to their actual visit. In line with Ball and colleagues recommendation for a study orientation, the use of virtual and recorded pre-enrollment messaging could serve to bolster recruitment in underserved rural areas where face-to-face information sessions may be less accessible to families (12). In addition to the video, CAB and PAT members identified sensitive language (e.g., obesity) in recruitment materials that elicited concern and sometimes offense by the targeted population. Likewise, CAB and PAT members worked together with the IRB to find compromises that satisfied institutional requirements while addressing participant concerns. Thus, developing feedback loops among academic partners, community stakeholders, and the targeted population is a key element in identifying and addressing issues of study participant trust and acceptance.

*Lesson 7. Develop systems to assess participant barriers to engagement at enrollment and periodically throughout the study to prepare to address target population obstacles*. Data from interviews with CAB and PAT partners and parent study participants indicated an underestimation of recruitment and engagement barriers faced by our participants. Research indicates that best practices around accessibility include (1) continuous individual assessment and support for attendance, (2) proximal sites, (3) incentives, and (4) flexible time options (15, 40, 44, 47). All of these practices were applied throughout our study. However, enrollment rates continued to decline and engagement remained at moderate levels. Protocols for re-engagement were often stymied by an inability to reach these participants and further understand and help address their barriers. These findings indicate more complex barriers to recruitment and engagement that require multi-level strategies with potential to incorporate alternate implementation and attendance options, such as asynchronous use of technology, mobile sites, and drop-in attendance. Because our study did not develop formal protocols to assess participant needs prior to enrollment and throughout the intervention, there were missed opportunities to identify specific engagement barriers and find novel approaches to support family commitment to the intervention (46, 51, 52).

*Lesson 8. Actively engage community partners in promoting readiness of the intended audience to engage in a childhood obesity treatment study*. CAB and PAT partners suggested that researchers should go beyond strategizing recruitment and engagement protocols and work to *promote* participant readiness. Childhood obesity awareness campaigns have successfully targeted physicians (53) and families (54) for engagement in programming. Providing physicians and families with tools to help parents identify their child's weight status, educate on contributing factors, prioritize treatment as a community need, and promote awareness of clinical trials to meet those needs could increase receptiveness to enrolling in healthy lifestyle change programs. This could prove an important preliminary step to conducting CER trials, particularly in an underserved, rural area like the DRR.

### Study Limitations and Strengths: Interpreting Multiple Perspectives

Our case study retrospectively evaluated stakeholder and participant interviews for recruitment and engagement indicators that were not explicitly intended by the interview protocols. Consequently, direct questions about recruitment and engagement were not consistently asked in the same manner over time or across interviewee groups. Furthermore, parent study participant data was only gathered from retained participants at follow-up assessments and does not include perspectives on recruitment and engagement from families that completely disengaged. Therefore, quantified analysis of the qualitative data should be interpreted cautiously when examining differences among key stakeholder responses. This approach to the data was meant to provide insight into the importance of multiple perspectives in painting a holistic picture of assets, challenges, and opportunities surrounding recruitment and engagement within the study. Due to the above mentioned methodological constraints, frequency of themes cannot suggest relative importance of one asset, challenge, or recommendation over another. However, a frequently mentioned theme may indicate something that is obvious to one or more of these interviewee groups and therefore provides insight into priorities and how they might fit together.

When examining the frequencies reported by the four interviewee groups, it is clear that a single perspective approach would have resulted in less complete and meaningful feedback on recruitment and engagement strategies within the study. For instance, enrolled parent study participants emphasized their readiness to participate in a childhood obesity treatment study and their positive perceptions of the interventions' acceptability. However, CAB and PAT members who experienced the recruitment and engagement efforts understood barriers for the larger target population. Likewise, while academic CAB members appeared to conclude that mistrust and discomfort with research hindered recruitment, community CAB members saw this as more of a mismatch between research and its ability to address community needs. Yet, parent study participants indicated research mistrust was largely due to randomization procedures. Furthermore, community partners and parent study participants were more inclined to understand intervention accessibility barriers than academic partners not living in the region.

In addition to providing a more holistic picture as to the assets, challenges, and opportunities related to recruitment and engagement, frequency patterns also suggested small nuances in the priorities of the interviewee groups. For instance, academic partners wished to improve the health of the targeted population. Additionally they wished to develop systems to continue research efforts with established partners. Thus, while community CAB and PAT partners were aware of the challenges related to building partner capacity at the organizational/systems level, these challenges may have been perceived more keenly by academic partners. Similarly, parent study participants' focus on assets and challenges related to the accessibility and acceptability of the interventions may suggest that they prioritized finding resources to meet their immediate family needs. Overall, the consistent approach of examining the data for prevalence of themes across stakeholders provided rich content information directly relevant to the strengths and pitfalls of our study. These findings may be relevant to other practitioners and researchers who work with populations that face numerous barriers to participation in research.

### Conclusion

Recruitment and engagement are perhaps the highest hurdles to overcome for FBCO and other interventions examining health behavior change, particularly for those engaging underserved, rural populations (15, 16). Our case study approach in examining recruitment and engagement through the lenses of various stakeholders provides insights regarding participant and stakeholder/organization needs in these areas. Findings highlighted the promise of CBPR approaches to bolster recruitment and engagement in hard to reach populations. They also highlighted the need for studies to include more in-depth tracking and examination of recruitment and engagement processes and to prioritize dissemination of these evaluations. Including multiple perspectives expanded understanding of assets and challenges and provided multi-leveled recommendations that could prove helpful to future studies targeting underserved, rural communities.

## Data Availability Statement

The raw data supporting the conclusions of this article will be made available by the authors, without undue reservation.

## Ethics Statement

The studies involving human participants were reviewed and approved by Institutional Review Board for Health Sciences Research University of Virginia. The patients/participants provided their written informed consent to participate in this study.

## Author Contributions

JZ and PE designed the overall study and methodology with substantial input from D-JB and JH. The focus of the manuscript was conceived by D-JB and JZ with support from PE, MY, and JH. Data analysis for this study was completed by D-JB, MY, and BP under the supervision of D-JB. As community partners in this project, JW, DM, and KE provided feedback on content interpretation. D-JB and JZ drafted the paper with section contributions from MY and editing from MY, PE, and JH. All authors provided feedback on the manuscript draft and approved the final version.

## Conflict of Interest

The authors declare that the research was conducted in the absence of any commercial or financial relationships that could be construed as a potential conflict of interest.
