# Supplementary material for: Assets and Challenges to Recruiting and Engaging Families in a Childhood Obesity Treatment Research Trial: Insights From Academic Partners, Community Partners, and Study Participants
Source: Front Public Health. 2021 Feb 22;9:631749. doi: 10.3389/fpubh.2021.631749 (PMC7937718; doi:10.3389/fpubh.2021.631749)
Supplement: Supplementary file 1 [file Table_1.DOCX]

**Supplementary Table 1. Assets and challenges to stakeholder/organization and participant level recruitment and engagement needs as reported by Academic CAB members (A), Community CAB members (C), PAT members (P), and Parent Study Participants (S)**

| **A**  **n=5** | **C**  **n=9** | **P**  **n=7** | **S**  **n=100** | **Stakeholder/Organization Level: Assets and Challenges** |
| --- | --- | --- | --- | --- |
|  |  |  |  | 1. ***Improve readiness of the stakeholders to conduct CBPR research*** |
| **100%** | **78%** | **71%** | **0%** | **Assets** |
| **X** | **X** | **X** |  | -Dedication and passion to improve the health of the community |
| **X** | **X** | **X** |  | -Confidence in ability to follow through on responsibilities |
|  |  |  |  | *“I think we know at this point that our Parks & Rec partners are always going to show up and…do what we ask them to do… They're going to show up [and]…do their job.”-A* |
| **100%** | **89%** | **86%** | **0%** | **Challenges** |
| **X** | **X** | **X** |  | -Incomplete milestones impacts confidence, study validity, and sustainability |
| **X** | **X** |  |  | -Research priorities/language/processes are unfamiliar to community partners |
| **X** | **X** |  |  | -Inequities in power/influence between CAB members |
| **X** | **X** |  |  | -Disparities in partner commitment to the study |
| **X** | **X** | **X** |  | -Navigation of research regulations of the IRB and the funder |
| **X** | **X** |  |  | -Turnover in CAB and PAT membership |
| **X** | **X** |  |  | -Turf issues between partners |
|  |  | **X** |  | -Lack of partner willingness to role model healthy lifestyles |
|  |  |  |  | *“The challenging thing is, as we expanded [efforts]…we really didn't see an increase in… [enrollment] that we thought we might. So it was…challenging emotionally…on staff.”-C* |
|  |  |  |  | 1. ***Develop sustainable participant referral protocols*** |
| **80%** | **100%** | **71%** | **13%** | **Assets** |
| **X** | **X** | **X** |  | -Flexibility/adaptability of PAT and CAB members in recruitment approaches |
| **X** | **X** | **X** |  | -Existing clinical partners capable of referring participants |
| **X** | **X** | **X** |  | -PAT and community partners willingness to take on leadership roles |
| **X** | **X** |  | **X** | -Families perceive physician referrals as legitimate |
| **X** | **X** |  |  | -Clear communication of roles and responsibilities |
|  |  |  |  | *“…we have the…[PAT] that are out there trying to encourage people into the program …and they're out there [recruiting in ways] we can't because we haven't actually done the program and they [have].”-C* |
| **100%** | **89%** | **100%** | **0%** | **Challenges** |
| **X** | **X** |  |  | -Lack of preparation for potential barriers |
| **X** | **X** | **X** |  | -Lack of metrics to determine the success of recruitment strategies |
| **X** | **X** | **X** |  | -Limited time and staffing of clinical partners |
| **X** | **X** |  |  | -Lack of follow through on tasks by Clinical Partners and PAT |
| **X** | **X** |  |  | -Intermittent workflow |
| **X** | **X** | **X** |  | -Expended referral pools |
| **X** | **X** |  |  | -Unestablished channels of communication between meetings |
|  |  | **X** |  | -PAT members that feel cut out of recruitment |
|  |  |  |  | *“I think in the beginning [the Clinical Partners] had every intention of [making screening calls], but because of circumstances... what they thought they could do, they were not able to do.”-A* |
|  |  |  |  | 1. ***Develop feasible/sustainable participant engagement protocols*** |
| **100%** | **78%** | **100%** | **0%** | **Assets** |
| **X** | **X** | **X** |  | -PAT and community partners willingness to take on leadership |
| **X** | **X** | **X** |  | -Data and observations indicate success of engagement strategies |
|  | **X** | **X** |  | -Development of back-up plans for carrying out engagement strategies |
| **X** |  | **X** |  | -Training and experience implementing engagement strategies |
|  |  |  |  | *“I love working with those parks and recs guys! They're just so good. They want to have an impact with the families…and they're great contributors in how we improve and focus on moving the project forward in a way that more families will benefit.”-A* |
| **100%** | **89%** | **100%** | **0%** | **Challenges** |
| **X** | **X** | **X** |  | - Complex logistics/limited resources impede engagement strategy implementation |
| **X** | **X** | **X** |  | -Lack of metrics to determine the success of engagement strategies |
| **X** | **X** | **X** |  | -Lack of follow through on tasks |
|  |  | **X** |  | -Limited leadership opportunities in engagement strategies |
|  |  |  |  | *“Our study wasn't designed to ask that question, did [an engagement strategy] work or not? [The PAT] was yet another piece of our engagement strategy. And we know overall our engagement was lower than what we would like...”-A* |
| **A**  **n=5** | **C**  **n=9** | **P**  **n=7** | **S**  **n=100** | **Participant Level: Assets and Challenges** |
|  |  |  |  | 1. ***Improve target population’s level of comfort and trust with research*** |
| **20%** | **67%** | **0%** | **1%** | **Assets** |
| **X** | **X** |  | **X** | -Study addresses a community need |
|  |  |  |  | *“I think the program that we're bringing to the table is giving families an opportunity to come and learn about nutrition and physical activity because I don't really think that we have many options here…” - C* |
| **40%** | **11%** | **0%** | **8%** | **Challenges** |
| **X** | **X** |  | **X** | -Target population’s distrust of research procedures |
|  |  |  |  | *“Think about a family who may not be familiar with research… and [they] get an invitation to a [study where] they're going to be randomized. There's a bit of mistrust in that process versus you know exactly what you're signing on to.” - A* |
|  |  |  |  | 1. ***Improve target population’s accessibility to the intervention*** |
| **20%** | **44%** | **14%** | **1%** | **Assets** |
| **X** | **X** | **X** | **X** | -Participation fit into busy family schedules (i.e., conveniently located and timed) |
|  |  |  |  | *“We thought that we’d get more participation by moving the facility to the city auditorium because it is centrally located and there is a bus hub not even a block away.” -P* |
| **60%** | **67%** | **71%** | **73%** | **Challenges** |
| **X** | **X** | **X** | **X** | -Balancing family priorities and limited time |
| **X** | **X** | **X** | **X** | -Unexpected life events |
| **X** | **X** | **X** | **X** | -Geographical distances and lack of transportation |
|  |  |  |  | *“Even though we always reached out to people in Pittsylvania County… a lot of them will say no when they realize what the travel times means…for their family.”-A* |
|  |  |  |  | 1. ***Improve target population’s awareness and understanding of the study*** |
| **80%** | **89%** | **71%** | **0%** | **Assets** |
| **X** | **X** | **X** |  | -Stakeholder/participant input on recruitment material and strategy modifications |
| **X** | **X** | **X** |  | -Shared decision making in the development of recruitment materials |
|  |  |  |  | *“[Recruitment materials] get passed to the parent advisory team. They have been through the program before and have gotten these calls and letters…so they know how it feels. So they go through it and they change a lot and then it goes back to the research team.”-C* |
| **80%** | **78%** | **57%** | **0%** | **Challenges** |
| **X** | **X** | **X** |  | -Low health literacy of target population |
| **X** | **X** | **X** |  | -Target population is difficult to reach |
| **X** | **X** | **X** |  | -Ineffective marketing strategies to target the intended population |
| **X** | **X** |  |  | -IRB regulations on recruitment procedures and language within materials |
|  |  |  |  | *“I guess research again! We sent out letters to a lot of families that we were targeting…to capture their attention. But … I think better marketing and more common language instead of a researcher's language would [increase] attraction of the programs.”-P* |
|  |  |  |  | 1. ***Improve target population’s acceptance of the intervention*** |
| **100%** | **78%** | **86%** | **92%** | **Assets** |
|  | **X** | **X** | **X** | -Intervention perceived as helpful, supportive, enjoyable, and informative by families |
|  |  |  | **X** | -Experienced and engaging intervention facilitators |
|  |  |  | **X** | -Intervention offers family time |
|  |  |  | **X** | -Family satisfied with the intervention they were randomized into |
| **X** | **X** | **X** |  | -Partner knowledge of the target population and experience with the intervention |
| **X** | **X** | **X** |  | -Curriculum provides hands on learning and goal tracking |
|  |  |  |  | *“[The facilitators] are really good at keeping you motivated and keeping children's attention. [They] make it fun for kids.”-S* |
| **20%** | **11%** | **57%** | **48%** | **Challenges** |
| **X** |  |  | **X** | -Dissatisfaction with randomization |
|  |  |  | **X** | -Dissatisfaction with the intervention components |
|  |  |  | **X** | -Anxiety over unintended consequences of enrolling |
|  |  |  | **X** | -Unsuitable/uncomfortable facilities |
|  |  | **X** |  | -Balancing intervention dosage with family needs for making changes |
|  | **X** | **X** |  | -Planned activities that rely on high levels of class attendance |
|  |  |  | **X** | -Family’s lack of progress in meeting goals |
|  |  |  |  | *“If child is overweight, he needs to be in class as well, not just the parent; I wanted my child to participate.”-S* |
|  |  |  |  | 1. ***Improve target population’s readiness to engage in an obesity treatment study*** |
| **20%** | **22%** | **14%** | **72%** | **Assets** |
|  | **X** |  | **X** | -Enrolled families are interested in their health, aware of their habits, and desire change |
| **X** |  | **X** | **X** | -Use of incentives motivates some families to enroll |
|  |  |  |  | *“I’m a diabetic and I don’t want her [my daughter] to end up like me. I just want her to be healthy.”-S* |
| **60%** | **78%** | **100%** | **18%** | **Challenges** |
| **X** | **X** | **X** | **X** | -Difficulty building and maintaining momentum for changing weight-related behaviors |
| **X** | **X** | **X** |  | -Lack of awareness of childhood obesity crisis |
| **X** | **X** |  |  | -Parent denial of their child’s weight status |
|  |  |  | **X** | -Family member disagreement over value of healthy lifestyle changes |
|  |  |  | **X** | -Culture that does not prioritize health and resists making lifestyle changes |
|  | **X** | **X** |  | -Incentives motivate families to enroll, but not to always stay |
|  |  |  |  | *“Yeah we have a lot of parents that are in denial. And we have to…figure out how to communicate with the[se] parents and… figure out the language [to] tell these parents that are qualified that their child is obese. “-C* |
